# Sex Differences in Oral Anticoagulation Therapy in Patients Hospitalized With Atrial Fibrillation: A Nationwide Cohort Study

**DOI:** 10.1161/JAHA.122.027211

**Published:** 2023-02-27

**Authors:** Kuan Ken Lee, Dimitrios Doudesis, Rong Bing, Federica Astengo, Jesus R. Perez, Atul Anand, Shauna McIntyre, Nicholas Bloor, Belinda Sandler, Steven Lister, Kevin G. Pollock, Ayesha C. Qureshi, David A. McAllister, Anoop S. V. Shah, Nicholas L. Mills

**Affiliations:** ^1^ BHF Centre for Cardiovascular Science University of Edinburgh Edinburgh United Kingdom; ^2^ Usher Institute of Population Health Sciences and Informatics, University of Edinburgh Edinburgh United Kingdom; ^3^ Institute of Health and Wellbeing, University of Glasgow Glasgow United Kingdom; ^4^ Bristol Myers Squibb Pharmaceuticals Ltd London United Kingdom; ^5^ Pfizer Ltd Tadworth United Kingdom; ^6^ London School of Hygiene and Tropical Medicine London United Kingdom

**Keywords:** atrial fibrillation, oral anticoagulation therapy, sex differences, Atrial Fibrillation, Ischemic Stroke, Secondary Prevention

## Abstract

**Background:**

Important disparities in the treatment and outcomes of women and men with atrial fibrillation (AF) are well recognized. Whether introduction of direct oral anticoagulants has reduced disparities in treatment is uncertain.

**Methods and Results:**

All patients who had an incident hospitalization from 2010 to 2019 with nonvalvular AF in Scotland were included in the present cohort study. Community drug dispensing data were used to determine prescribed oral anticoagulation therapy and comorbidity status. Logistic regression modeling was used to evaluate patient factors associated with treatment with vitamin K antagonists and direct oral anticoagulants. A total of 172 989 patients (48% women [82 833 of 172 989]) had an incident hospitalization with nonvalvular AF in Scotland between 2010 and 2019. By 2019, factor Xa inhibitors accounted for 83.6% of all oral anticoagulants prescribed, while treatment with vitamin K antagonists and direct thrombin inhibitors declined to 15.9% and 0.6%, respectively. Women were less likely to be prescribed any oral anticoagulation therapy compared with men (adjusted odds ratio [aOR], 0.68 [95% CI, 0.67–0.70]). This disparity was mainly attributed to vitamin K antagonists (aOR, 0.68 [95% CI, 0.66–0.70]), while there was less disparity in the use of factor Xa inhibitors between women and men (aOR, 0.92 [95% CI, 0.90–0.95]).

**Conclusions:**

Women with nonvalvular AF were significantly less likely to be prescribed vitamin K antagonists compared with men. Most patients admitted to the hospital in Scotland with incident nonvalvular AF are now treated with factor Xa inhibitors and this is associated with fewer treatment disparities between women and men.

**Nonstandard Abbreviations and Acronyms**
ATC: Anatomical Therapeutic Chemical classification system
BARC: Bleeding Academic Research Consortium
CCI: Charlson Comorbidity Index
DOAC: direct oral anticoagulant
GRO: General Register of Scotland
MACE: major adverse cardiovascular events
NHS: National Health Service
OPCS: Office of Population Censuses and Surveys
PIS: Prescribing Information System
SIMD: Scottish Index of Multiple Deprivation
SMR01: Scottish Morbidity Record 01
TTR: time in therapeutic range
WHO: World Health Organization

**Clinical Perspective**

**What Is New?**

- In a contemporary nationwide cohort study using individual patient data linkage, more than one‐third of patients admitted to the hospital in Scotland with incident nonvalvular atrial fibrillation remained untreated with any oral anticoagulation.
- Women were less likely to receive oral anticoagulation therapy than men, and the disparity was primarily attributable to vitamin K antagonists, while there was less disparity in treatment with direct factor Xa inhibitors.
- Women who were not treated with oral anticoagulation therapy experienced the highest rates of subsequent major adverse cardiovascular events and all‐cause mortality, with no significant difference in rates of bleeding.

**What Are the Clinical Implications?**

- Most patients admitted to the hospital with incident nonvalvular atrial fibrillation are now treated with direct factor Xa inhibitors and this is associated with fewer treatment disparities between women and men and in older, more comorbid patients.
- If these trends continue, disparities in oral anticoagulation therapy between women and men with atrial fibrillation may be eliminated through increased treatment with direct factor Xa inhibitors.

Women are at increased risk of ischemic stroke caused by atrial fibrillation (AF); therefore, current clinical practice guidelines recommend use of risk stratification scores that incorporate female sex to guide use of oral anticoagulation therapy.^1^, ^2^, ^3^, ^4^ Despite this guidance, women with AF remain undertreated and their outcomes are poorer compared with men.^5^, ^6^

Vitamin K antagonists, mainly warfarin, have been the mainstay for stroke prophylaxis in patients with AF for many decades, but, in the past few years, direct oral anticoagulants (DOACs) have been introduced into clinical practice as alternatives. The safe use of warfarin requires careful dose titration to maintain patients within a narrow therapeutic range of international normalized ratio (INR), but this is challenging, with many registries demonstrating suboptimal time in therapeutic range (TTR), resulting in higher rates of mortality, major bleeding, and stroke.^7^, ^8^ Conversely, all DOACs have a predictable therapeutic effect without need for dose titration and regular anticoagulation monitoring. In a meta‐analysis of randomized controlled trials, DOACs were reported to be more effective compared with warfarin in preventing strokes or mortality.^9^ It is not known whether the introduction of the DOACs has reduced disparities in treatment between women and men with AF.

In this study, we aimed to evaluate the trends in oral anticoagulation prescribing for women and men admitted to the hospital with nonvalvular AF and compare the factors influencing the prescription of vitamin K antagonist and DOACs.

## METHODS

This study makes use of routine electronic health care data sources that are linked, deidentified, and held in the National Health Service (NHS) national safe haven, which is accessible by approved individuals who have undertaken the necessary governance training. Summary data can be made available on request to the corresponding author.

### Study Design and Data Sources

We linked multiple national databases to conduct this nationwide cohort study. We identified all patients aged 18 years or older who were admitted to the hospital in Scotland with nonvalvular AF between January 1, 2010, and December 31, 2019, from the Scottish Morbidity Record (SMR01) held by Public Health Scotland. Individual patient episodes were linked to the national drug prescribing database held by the Prescribing Information System (PIS) and the General Register of Scotland (GRO), which contains information on all in‐hospital and community deaths in the country.^10^ Access to the data was approved by the Privacy Advisory Committee of the NHS Scotland Public Benefit and Privacy Panel for Health and Social Care and in accordance with the Declaration of Helsinki. As all data used in this analysis had already been collected and anonymized, individual patient consent was not required or sought.

### Patient Population

Incident admissions to the hospital with AF were identified from hospital discharge records within the SMR01 database using codes from the *International Classification of Diseases, Tenth Revision* (*ICD‐10*). We used the *ICD‐10* code I48, which includes both AF and atrial flutter but does not include other forms of atrial arrhythmias. A 10‐year look‐back period was used to exclude any recurrent hospitalizations of the same individual patient (Data S1).^11^ Patients with previous mitral valve surgery or on hemodialysis were excluded from this analysis because DOACs are currently contraindicated for these patients.

### Definition of Covariates

Demographic information for each individual patient was obtained from the SMR01 database. We have defined patients' sex as the self‐reported sex documented in the SMR01 database. We used the Scottish Index of Multiple Deprivation (SIMD), an area‐based measure of deprivation, to define socioeconomic status of each individual. The SIMD 2009 combines 31 indicators across 7 domains: income, employment, health, education, health, crime and housing, and access to services.^12^ The overall SIMD is a weighted sum of the 7 domain scores across 6976 small areas (called data zones). These data zones are then ranked by SIMD quintile from most deprived (first quintile) to least deprived (fifth quintile).

Patient comorbidities were defined using information from previous hospitalizations, hospital procedures, and drug prescribing data. We categorized prescribed drugs using the World Health Organization (WHO) Anatomical Therapeutic Chemical (ATC) classification system to define comorbid conditions. Using these comorbid conditions, we calculated each patient's CHA_2_DS_2_VASc score to define their individual risk of thromboembolic complications.^13^ The full details on the hospital episode and ATC codes to define each comorbid condition are presented in Data S2. Using the same methodology, we calculated the Charlson Comorbidity Index (CCI) for each individual patient to assess burden of comorbidity. The CCI includes 19 conditions. Each condition is assigned a weight from 1 to 6 and summed to produce the score (range 1–37).

### Outcomes

Bleeding Academic Research Consortium (BARC) (Data S3).^14^ These codes have been validated using linked bespoke studies and electronic health records by the CALIBER group.^15^

### Statistical Analysis

Baseline characteristics of women and men with incident hospitalization with nonvalvular AF were described. Categorical variables were described as proportions, while means and SDs were reported for continuous variables. We described trends in oral anticoagulation prescription for women and men hospitalized with incident nonvalvular AF between 2010 and 2019. In patients with thromboembolic risk factors (CHA_2_DS_2_VASc score >0 in men and >1 in women), we used a logistic regression model to evaluate the association between important patient factors such as age, sex, deprivation, burden of comorbidity, prior bleeding and thromboembolic risk, and prescription of oral anticoagulants. We initially performed these analyses for all oral anticoagulants and subsequently stratified by vitamin K antagonists and direct factor Xa inhibitors. We also constructed cumulative incidence functions to describe of women and men stratified by whether they were prescribed oral anticoagulation following the incident hospitalization with nonvalvular AF. We applied competing risk methodology to estimate the cumulative incidence function of ischemic stroke, MI, and bleeding to account for the competing risk of all‐cause mortality.^16^ All statistical analyses were performed in R version 3.6.1 (The R Foundation).

## RESULTS

Overall, 172 989 patients had an incident hospitalization with nonvalvular AF in Scotland over the 10‐year period between 2010 and 2019, of whom, 82 833 (48%) were women and 90 156 (52%) were men. Women presenting with nonvalvular AF were older than men (78±11 years versus 73±13 years) and had a higher CHA_2_DS_2_VASc score (4.1±1.3 versus 2.8±1.5). Compared with men, women had a similar prevalence of previous heart failure, hypertension, ischemic stroke, and prior bleeding, but a lower prevalence of diabetes and previous MI (Table).

**Table 1:** Characteristics of Patients Hospitalized With AF Stratified by Sex

	Overall	Men	Women
No. of patients	172 989	90 156	82 833
Age, y	75.3 (12.1)	72.7 (12.5)	78.1 (11.1)
Previous medical conditions			
MI	7800 (4.5)	4646 (5.2)	3154 (3.8)
Stroke	3792 (2.2)	1824 (2.0)	1968 (2.4)
Heart failure	10 989 (6.4)	5941 (6.6)	5048 (6.1)
Previous coronary revascularization	7349 (4.2)	5411 (6.0)	1938 (2.3)
Hypertension	98 583 (57.0)	51 624 (57.3)	46 959 (56.7)
Chronic lower respiratory disease	39 335 (22.7)	19 175 (21.3)	20 160 (24.3)
Diabetes	23 803 (13.8)	14 016 (15.5)	9787 (11.8)
Previous bleeding	9599 (5.5)	5102 (5.7)	4497 (5.4)
CHA_2_DS_2_VASc score	3.4 (1.5)	2.8 (1.5)	4.1 (1.3)
SIMD quintile			
1 (most deprived)	34 224 (20.1)	16 927 (19.1)	17 297 (21.1)
2	37 183 (21.8)	18 538 (20.9)	18 645 (22.8)
3	35 326 (20.7)	18 583 (20.9)	16 743 (20.5)
4	33 569 (19.7)	18 267 (20.6)	15 302 (18.7)
5 (least deprived)	30 380 (17.8)	16 534 (18.6)	13 846 (16.9)
CCI			
0	113 722 (65.7)	59 032 (65.5)	54 690 (66.0)
1	36 964 (21.4)	18 899 (21.0)	18 065 (21.8)
2	15 116 (8.7)	8210 (9.1)	6906 (8.3)
≥3	7187 (4.2)	4015 (4.5)	3172 (3.8)

Values are presented as number (percentage). Scottish Index of Multiple Deprivation (SIMD) combines 31 indicators across 7 domains: income, employment, health, education, health, crime and housing, and access to services. The overall SIMD is ranked by SIMD quintile from most deprived (first quintile) to least deprived (fifth quintile). A higher Charlson Comorbidity Index (CCI) indicates greater comorbidity burden. AF indicates atrial fibrillation; and MI, myocardial infarction.

**Figure 1:**
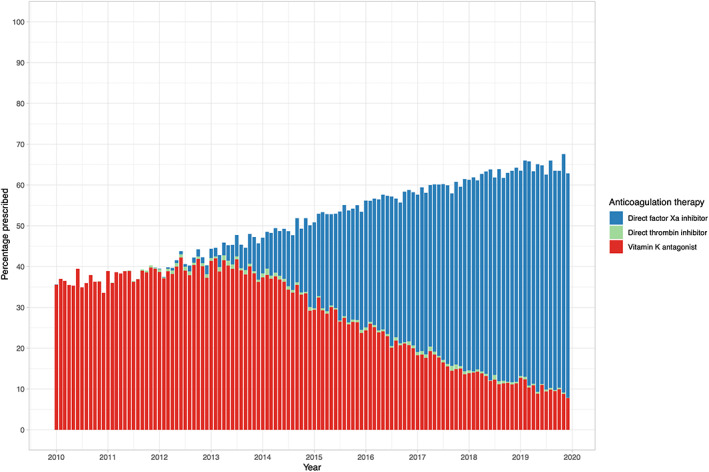
Trends in oral anticoagulation therapy for patients admitted to the hospital with nonvalvular atrial fibrillation in Scotland from 2010 to 2019.

Approximately half of all patients hospitalized with nonvalvular AF were prescribed oral anticoagulation therapy (51.3% [88 828/172 989]). The proportion of patients prescribed oral anticoagulation therapy increased from 36.2% in 2010 to 64.5% in 2019 (Figure 1). In patients with thromboembolic risk factors (CHA_2_DS_2_VASc score >0 in men and >1 in women), the proportion prescribed oral anticoagulation therapy was marginally higher (36.8% in 2010 and 66.3% in 2019) (Figure S1). Patients with thromboembolic risk factors who were not prescribed any oral anticoagulation therapy were of similar age (78±11 years versus 76±10 years) and had a similar CHA_2_DS_2_VASc score (3.6±1.4 versus 3.7±1.3) but were more likely to be women (51.8% versus 46.8%) compared with those who received oral anticoagulation therapy (Table S1). Throughout the study period, a lower proportion of women compared with men were prescribed oral anticoagulation (33% versus 41% in 2010 and 65% versus 68% in 2019) (Figure S2). In a multivariable logistic regression model adjusted for age, deprivation, comorbidity, prior bleeding, and CHA_2_DS_2_VASc score, women were significantly less likely to receive oral anticoagulation therapy compared with men (adjusted odds ratio [aOR], 0.68 [95% CI, 0.67–0.70]).

**Figure 2:**
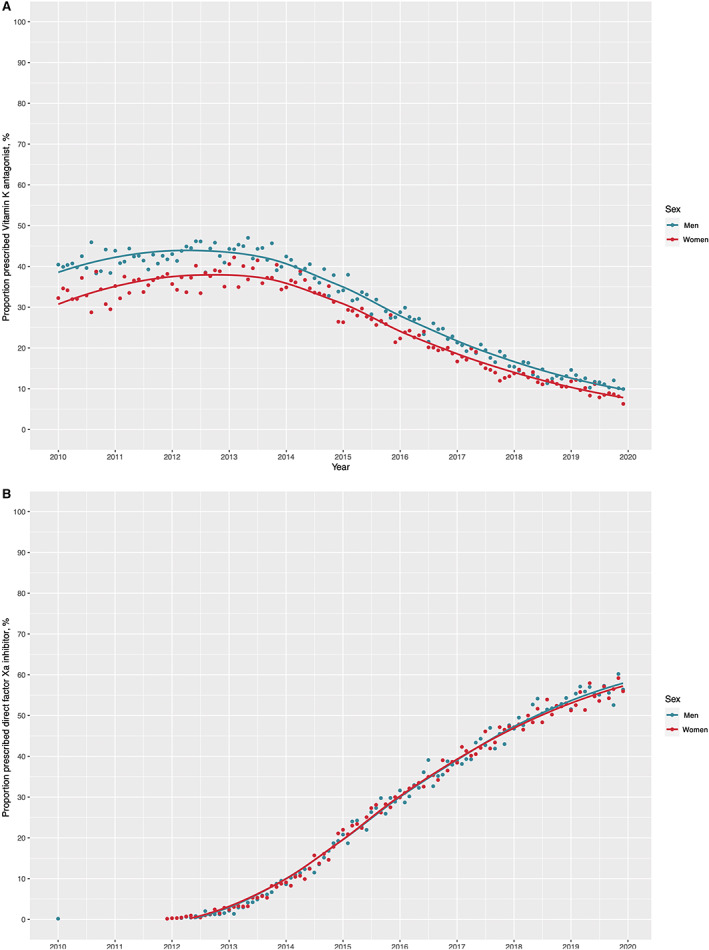
Trends in oral anticoagulation therapy for patients admitted to the hospital with nonvalvular atrial fibrillation in Scotland from 2010 to 2019 with thromboembolic risk factors (CHA_2_DS_2_VASc score >0 in men and >1 in women) stratified by sex. **A**, Trends in vitamin K antagonist prescribing. **B**, Trends in factor Xa inhibitor prescribing.

**Figure 3:**
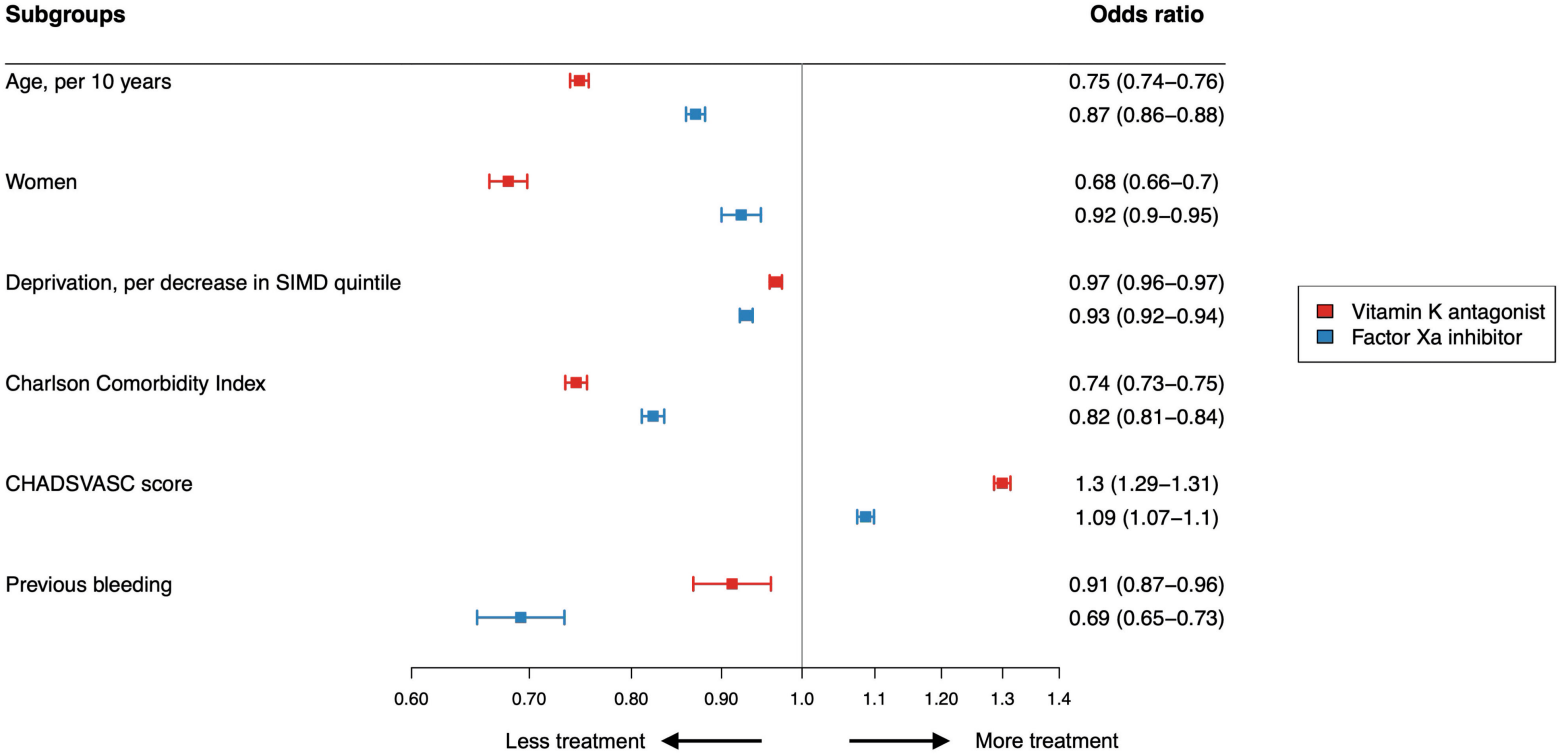
Factors associated with vitamin K antagonist and factor Xa inhibitor therapy in patients admitted to the hospital with nonvalvular atrial fibrillation with thromboembolic risk factors (CHA_2_DS_2_VASc score >0 in men and >1 in women). Odds ratios were derived from multivariable logistic regression models including age, sex, deprivation (Scottish Index of Multiple Deprivation [SIMD]), burden of comorbidity (Charlson Comorbidity Index), prior bleeding, and thromboembolic risk (CHA_2_DS_2_VASc score).

Of those with thromboembolic risk factors who were prescribed oral anticoagulation therapy, 53.5% (45 318/84764) were prescribed vitamin K antagonists, while 45.5% (38 583/84 764) were prescribed direct factor Xa inhibitors and 1.0% (863/84 764) were prescribed direct thrombin inhibitors. Treatment with vitamin K antagonists declined from 100% of all oral anticoagulation therapy prescribed in 2010 to 15.9% in 2019 (Figure 1). Meanwhile, use of direct factor Xa inhibitors has increased from 0% to 83.6%. Direct thrombin inhibitors peaked in 2013 at 1.7%, but this decreased to 0.6% in 2019. Throughout the study period, women were less likely to be prescribed vitamin K antagonists compared with men (33% versus 41% in 2010 and 9% versus 12% in 2019) (Figure 2A). Conversely, the proportion of women and men prescribed direct factor Xa inhibitors was similar (Figure 2B). In multivariable logistic regression modeling, women were significantly less likely to receive vitamin K antagonists compared with men (aOR, 0.68 [95% CI, 0.66–0.70]). Older patients and those with comorbidities were also less likely to receive vitamin K antagonists (aOR, 0.75 [95% CI, 0.74–0.76] per 10‐year increments in age and aOR, 0.74 [95% CI, 0.73–0.74] per unit increase in CCI score, respectively) (Figure 3). These observations were consistent in an additional post hoc analysis adjusting for thromboembolic risk factors where sex category was removed from the CHA_2_DS_2_VASc score (Figure S3). There were fewer disparities in the prescription of direct factor Xa inhibitors (aOR for women versus men 0.92 [95% CI, 0.90–0.95]); however, those with a history of prior bleeding were less likely to receive direct factor Xa inhibitors (aOR, 0.69 [95% CI, 0.65–0.73]).

Patients not prescribed oral anticoagulation therapy were more likely to have subsequent MACE compared with those prescribed oral anticoagulation therapy at 30 days (14.9% [5914 of 39 608] versus 4.2% [1669 of 39 671] in women and 13.0% [4801 of 36 868] versus 4.4% [1970 of 45 093] in men) and 1 year (38.8% [15 380 of 39 608] versus 17.0% [6761 of 39 671] in women and 35.2% [12 977 of 36 868] versus 16.4% [7395 of 45 093] in men). (Table S2). Women who were not prescribed oral anticoagulation had the highest rates of subsequent ischemic stroke, all‐cause mortality, and MACE compared with men who were or were not prescribed oral anticoagulation therapy, with no significant differences in incident major bleeding (Figure S4).

## DISCUSSION

In this contemporary nationwide cohort study using individual patient data linkage, we evaluated the trends and factors associated with oral anticoagulation prescribing for women and men admitted to the hospital in Scotland with nonvalvular AF and their subsequent risk of all‐cause mortality and bleeding over the past decade. We make several important observations. First, between 2010 and 2019, only half of all patients admitted with nonvalvular AF were prescribed any oral anticoagulation therapy, although this increased to nearly two‐thirds by 2019. Women were less likely than men to receive oral anticoagulation therapy even after accounting for age, comorbidities, thromboembolic risk factors, and deprivation. Second, the disparity in oral anticoagulation therapy between women and men was attributable to vitamin K antagonists, while treatment with direct factor Xa inhibitors was similar between women and men. Treatment with factor Xa inhibitors was also associated with a reduction in treatment disparities in older patients and those with more comorbidities; however, those with prior bleeding were significantly less likely to receive this class of anticoagulant. Finally, women who were not treated with oral anticoagulation therapy experienced the highest rates of subsequent MACE and all‐cause mortality with no significant difference in rates of bleeding.

Previous studies evaluating the association between sex and prescription of oral anticoagulation therapy have reported divergent findings. Several previous studies have reported similar rates of oral anticoagulation prescribing in women and men, while others have demonstrated that women were less likely to be prescribed oral anticoagulation.^17^, ^18^, ^19^, ^20^ Importantly, most of these data were derived from commercial insurance claims databases or patient registries that require individual patient consent and voluntary submission of data, which were likely to have introduced significant selection bias. In contrast, our study utilized a national database to identify all consecutive patients presenting to hospitals nationwide with incident AF without selection bias. Furthermore, most previous studies were conducted during the initial years after the introduction of DOACs into clinical practice when warfarin was the most widely prescribed oral anticoagulant. Here, we evaluated longer‐term trends in oral anticoagulation therapy over a decade after introduction of DOACs and observed that trends in prescribing continued to evolve over time. Finally, previous studies have mainly identified patients in primary care or in outpatient settings, whereas we have included patients admitted to secondary care with incident AF. These patients are significantly older, frailer, and have more comorbidities and severe complications from AF and it is in this patient population where there may be more uncertainty about the risks and benefits of anticoagulation therapy.

Despite clear national^21^ and international^1^ guidelines highlighting female sex as an important thromboembolic risk factor, we observed that women remain less likely to receive oral anticoagulation therapy than men. In Scotland, all medications prescribed in primary and secondary care within the NHS is paid for by the government. Differences in oral anticoagulation prescribing between women and men persisted even after adjusting for age, deprivation, comorbidities, and CHA_2_DS_2_VASc score. Our analysis suggests that much of this disparity was primarily attributable to warfarin, while there was no difference in DOAC prescribing between women and men. Consistent with multiple other registries, we observed a rapid increase in prescription of direct factor Xa inhibitors for patients with AF in recent years, with a concurrent decrease in warfarin prescription.^22^, ^23^, ^24^ Our data suggest that increased use of direct factor Xa inhibitors has, in effect, narrowed sex differences in oral anticoagulation prescribing in patients with AF over the past decade. It is therefore possible that if current trends in increased prescription of direct factor Xa inhibitor therapy continues, sex differences in oral anticoagulation therapy in patients with AF may be significantly reduced.

There are several reasons that may potentially explain sex differences in the prescription of warfarin and direct factor Xa inhibitors. Women may be more likely to decline warfarin therapy because of lack of social support or access to primary care to attend regular INR monitoring.^25^ Indeed, multiple studies on patients treated with warfarin have reported significantly lower TTR in women compared with men.^26^, ^27^, ^28^, ^29^ Difficulty in achieving a stable INR may have also influenced clinicians' prescribing decisions since low TTR is strongly associated with reduced efficacy and increased risk of adverse outcomes such as bleeding.^30^, ^31^ In contrast to warfarin, factor Xa inhibitors have a predictable therapeutic effect without the need for regular INR monitoring. Furthermore, multiple studies have demonstrated greater benefit of factor Xa inhibitors in patients with labile INR.^32^, ^33^ This important advantage may have encouraged more women and their clinicians to pursue oral anticoagulation therapy with factor Xa inhibitors, in particular, those who may otherwise have decided not to receive warfarin therapy.

We acknowledge several limitations in this study. Despite optimal adjustment for potential confounders, it is possible that disparities in prescription of oral anticoagulation therapy in women and men were attributable to unmeasured sex‐specific differences in baseline characteristics and we were unable to account for this residual confounding. Furthermore, we did not have access to detailed individual patient records; therefore, it is possible that a significant number of these patients had contraindications to anticoagulation therapy. However, it is unlikely that this is the case for most patients with nonvalvular AF in secondary care. While the prescribing database contains comprehensive data on all medications dispensed to individual patients, we do not have information on adherence to the medications. We also did not have information on individual patients' INR or TTR for patients taking warfarin. This may have introduced some exposure misclassification. Moreover, we did not have data on patients' sex. This may also be an important factor influencing prescribing decision‐making and outcomes in addition to biological sex. Finally, we relied on *ICD* diagnostic codes to identify our study population and define comorbidities and subsequent outcomes. We accept that there may be a degree of inaccuracy in the coding of these events.

## CONCLUSIONS

More than one‐third of patients admitted to the hospital in Scotland with incident nonvalvular AF remained untreated with any oral anticoagulation. Women are significantly less likely to be prescribed oral anticoagulation therapy compared with men, and this is mainly attributable to vitamin K antagonists. Most patients admitted to the hospital in Scotland with incident nonvalvular AF are now treated with direct factor Xa inhibitors and this is associated with fewer treatment disparities between women and men and in older, more comorbid patients. If these trends continue, disparities in oral anticoagulation prescribing between women and men with AF may be significantly reduced through increased treatment with factor Xa inhibitors.

## Sources of Funding

This study was supported by the British Heart Foundation through a Clinical Research Training Fellowship (FS/18/25/33454), Intermediate Clinical Research Fellowship (FS/19/17/34172), Senior Clinical Research Fellowship (FS/16/14/32023), and a Research Excellence Award (RE/18/5/34216). D.A.M. is funded by a Wellcome Trust Intermediate Clinical Fellowship (201492/Z/16/Z). This study was also supported by a research grant to LHS Lothian from Bristol Myers Squibb Pharmaceuticals Ltd and Pfizer UK Ltd.

## Disclosures

A.C.Q., K.G.P., S.L., S.M., and B.S. are employees of Bristol Myers Squibb Pharmaceuticals Ltd. N.B. is an employee of Pfizer UK Ltd.

## Supporting information

Data S1–S3
Tables S1–S2
Figures S1–S4
