# Supplementary material for: Sex Differences in Oral Anticoagulation Therapy in Patients Hospitalized With Atrial Fibrillation: A Nationwide Cohort Study
Source: J Am Heart Assoc. 2023 Feb 27;12(5):e027211. doi: 10.1161/JAHA.122.027211 (PMC10111444; doi:10.1161/JAHA.122.027211)

SUPPLEMENTAL MATERIAL

Data S1. Method for calculating 10-year look back period.

Any hospitalization after the start of the cohort period, where there was no previous event within 10 years is considered incident and summed for each calendar year. The tables below show a worked example for the calculation of incident hospitalizations for two patients included in this study.

Example case 1:

**Patient id**	**Episode id**	**Date**	**Diagnostic codes**					**Incident hospitalization**
			**Primary**	**Condition 1**	**Condition 2**	**Condition 3**	**Condition 4**	
1	1	01/1994	427.3	410				0
1	2	01/2010	I21	I50	I48			1
1	3	01/2013	I48					0

Example case 2:

**Patient id**	**Episode id**	**Date**	**Diagnostic codes**					**Incident hospitalization**
			**Primary**	**Condition 1**	**Condition 2**	**Condition 3**	**Condition 4**	
2	1	01/2010	429	410	427.3			1
2	2	01/2013	427.3					0
2	3	01/2015	I48					0

Data S2. Hospital and Anatomical Therapeutic Chemical (ATC) Classification codes used to define patient comorbidity.

Comorbidity	Definition	
	ATC	ICD-10
Atrial fibrillation		I48
Myocardial infarction		I21, I22
Ischemic stroke		I63
Heart failure		I11.0, I13.0, I13.2, I42.6, I50
Hypertension	C02, C09, C08ca, C03a, C03ba11	
Chronic lower respiratory disease	R03	
Diabetes mellitus	A10	

Data S3. Bleeding Academic Research Consortium Definition for Bleeding (BARC) [14]

Types	Definition
Type 0	no bleeding
Type 1	bleeding that is not actionable and does not cause the patient to seek unscheduled performance of studies, hospitalization, or treatment by a healthcare professional; may include episodes leading to self-discontinuation of medical therapy by the patient without consulting a healthcare professional
Type 2	any overt, actionable sign of haemorrhage (eg, more bleeding than would be expected for a clinical circumstance, including bleeding found by imaging alone) that does not fit the criteria for type 3, 4, or 5 but does meet at least one of the following criteria: (1) requiring nonsurgical, medical intervention by a healthcare professional, (2) leading to hospitalization or increased level of care, or (3) prompting evaluation
Type 3a	Overt bleeding plus haemoglobin drop of 3 to <5 g/dL (provided haemoglobin drop is related to bleed) or any transfusion with overt bleeding
Type 3b	Overt bleeding plus haemoglobin drop ≥5 g/dL (provided haemoglobin drop is related to bleed), cardiac tamponade, bleeding requiring surgical intervention for control (excluding dental/nasal/skin/haemorrhoid), or bleeding requiring intravenous vasoactive agents
Type 3c	Intracranial haemorrhage (does not include micro bleeds or haemorrhagic transformation, does include intraspinal), subcategories confirmed by autopsy or imaging or lumbar puncture, or intraocular bleed compromising vision
Type 4	CABG-related bleeding defined as perioperative intracranial bleeding within 48 hr, reoperation after closure of sternotomy for the purpose of controlling bleeding, transfusion of ≥5 U whole blood or packed red blood cells within a 48-h period, or chest tube output ≥2L within a 24-h period

Type 5a	Probable fatal bleeding; no autopsy or imaging confirmation but clinically suspicious
Type 5b	Definite fatal bleeding; overt bleeding or autopsy or imaging confirmation

Diagnostic codes to define bleeding events according to the Consensus Report from the Bleeding Academic Research Consortium (BARC) criteria.
(1) BARC 2 **(2)** BARC 3a **(3)** BARC 3b **(4)** BARC 3b or 4 if after CABG **(5)** BARC 3c
(6) BARC 3c or 4 if after CABG **(7)** BARC 4 **(8)** BARC 5

ICD code	ICD definition	category
K226	Gastro-oesophageal laceration-haemorrhage syndrome	1
K625	Haemorrhage of anus and rectum	1
K920	Haematemesis	1
K921	Melaena	1
K922	Gastrointestinal haemorrhage, unspecified	1
N837	Haematoma of broad ligament	1
N938	Other specified abnormal uterine and vaginal bleeding	1
N939	Abnormal uterine and vaginal bleeding, unspecified	1
O031	Spontaneous abortion ; Incomplete, complicated by delayed or excessive haemorrhage	1
O036	Spontaneous abortion; Complete or unspecified, complicated by excessive haemorrhage	1
O041	Medical abortion ; Incomplete, complicated by delayed or excessive haemorrhage	1
O046	Medical abortion ; Complete or unspecified, complicated by delayed or excessive haemorrhage	1
O051	Other abortion ; Incomplete, complicated by delayed or excessive haemorrhage	1
O056	Other abortion ; Complete or unspecified, complicated by delayed or excessive haemorrhage	1
O061	Unspecified abortion ; Incomplete, complicated by delayed or excessive haemorrhage	1
O066	Unspecified abortion ; Complete or unspecified, complicated by excessive haemorrhage	1
O071	Failed medical abortion, complicated by delayed or excessive haemorrhage	1
O076	Other and unspecified failed attempted abortion, complicated by excessive haemorrhage	1
O081	Delayed or excessive haemorrhage following abortion and ectopic and molar pregnancy	1
O208	Other haemorrhage in early pregnancy	1
O209	Haemorrhage in early pregnancy, unspecified	1

O46	Antepartum haemorrhage, not elsewhere classified	1
O717	Obstetric haematoma of pelvis	1
O902	Haematoma of obstetric wound	1
R042	Haemoptysis	1
T810	Haemorrhage and haematoma complicating a procedure, not elsewhere classified	1
K250	Gastric ulcer ; Acute with haemorrhage	2
K254	Gastric ulcer ; Chronic or unspecified with haemorrhage	2
K260	Duodenal ulcer ; Acute with haemorrhage	2
K264	Duodenal ulcer ; Chronic or unspecified with haemorrhage	2
K270	Peptic ulcer, site unspecified ; Acute with haemorrhage	2
K274	Peptic ulcer, site unspecified ; Chronic or unspecified with haemorrhage	2
K280	Gastrojejunal ulcer ; Acute with haemorrhage	2
K284	Gastrojejunal ulcer ; Chronic or unspecified with haemorrhage	2
K290	Acute haemorrhagic gastritis	2
O67	Labour and delivery complicated by intrapartum haemorrhage, not elsewhere classified	2
O720	Third-stage haemorrhage	2
O721	Other immediate postpartum haemorrhage	2
O722	Delayed and secondary postpartum haemorrhage	2
P261	Massive pulmonary haemorrhage originating in the perinatal period	2
R041	Haemorrhage from throat	2
R048	Haemorrhage from other sites in respiratory passages	2
R049	Haemorrhage from respiratory passages, unspecified	2
I850	Oesophageal varices with bleeding	3
K252	Gastric ulcer ; Acute with both haemorrhage and perforation	3
K256	Gastric ulcer ; Chronic or unspecified with both haemorrhage and perforation	3
K262	Duodenal ulcer ; Acute with both haemorrhage and perforation	3
K266	Duodenal ulcer ; Chronic or unspecified with both haemorrhage and perforation	3
K272	Peptic ulcer, site unspecified ; Acute with both haemorrhage and perforation	3
K276	Peptic ulcer, site unspecified ; Chronic or unspecified with both haemorrhage and perforation	3
K282	Gastrojejunal ulcer ; Acute with both haemorrhage and perforation	3
K286	Gastrojejunal ulcer ; Chronic or unspecified with both haemorrhage and perforation	3
H356	Retinal haemorrhage	5
H431	Vitreous haemorrhage	5
H450	Vitreous haemorrhage in diseases classified elsewhere	5
I60	Subarachnoid haemorrhage	5
I61	Intracerebral haemorrhage	5
I62	Other nontraumatic intracranial haemorrhage	5
I690	Sequelae of subarachnoid haemorrhage	5

I692	Sequelae of other nontraumatic intracranial haemorrhage	5
S064	Epidural haemorrhage	5
S065	Traumatic subdural haemorrhage	5
S066	Traumatic subarachnoid haemorrhage	5
I230	Haemopericardium as current complication following acute myocardial infarction	7
I312	Haemopericardium, not elsewhere classified	7

Table S1. Characteristics of patients hospitalized with atrial fibrillation with thromboembolic risk factors (CHA_2DS_2VASc score >0 in men and >1 in women) stratified by prescription of oral anticoagulation

	Overall	Prescribed anticoagulation	No anticoagulation
**Number of patients**	161240	84764	76476
**Age, years**	76.9 (10.5)	75.8 (9.9)	78.2 (11.0)
**Women**	79279 (49.2)	39671 (46.8)	39608 (51.8)
**Previous medical conditions**			
Myocardial infarction	7645 (4.7)	2971 (3.5)	4674 (6.1)
Stroke	3792 (2.4)	1960 (2.3)	1832 (2.4)
Heart Failure	10989 (6.8)	5069 (6.0)	5920 (7.7)
Previous coronary revascularisation	7205 (4.5)	2814 (3.3)	4391 (5.7)
Hypertension	98583 (61.1)	56134 (66.2)	42449 (55.5)
Chronic lower respiratory disease	37335 (23.2)	19291 (22.8)	18044 (23.6)
Diabetes mellitus	23803 (14.8)	13287 (15.7)	10516 (13.8)
Previous bleeding	9152 (5.7)	3751 (4.4)	5401 (7.1)
**CHA_2DS_2VASc score**	3.7 (1.3)	3.7 (1.3)	3.6 (1.4)
**SIMD quintile**			
1 (most deprived)	31701 (19.9)	15388 (18.3)	16313 (21.7)
2	34849 (21.9)	17736 (21.1)	17113 (22.8)
3	33024 (20.7)	17568 (20.9)	15456 (20.6)
4	31389 (19.7)	17550 (20.8)	13839 (18.4)
5 (least deprived)	28414 (17.8)	16009 (19.0)	12405 (16.5)
**Charlson Comorbidity Index**			
0	103940 (64.5)	60035 (70.8)	43905 (57.4)
1	35372 (21.9)	15868 (18.7)	19504 (25.5)
2	14779 (9.2)	6118 (7.2)	8661 (11.3)
≥3	7149 (4.4)	2743 (3.2)	4406 (5.8)

Presented as number of patients (%). Abbreviations: SIMD = Scottish index for multiple deprivation. SIMD combines 31 indicators across 7 domains: income, employment, health, education, health, crime and housing, and access to services. The overall SIMD index are ranked by SIMD quintile from most deprived (1st quintile) to least deprived (5th quintile). A higher Charlson Comorbidity index indicates greater comorbidity burden.

Table S2. Outcomes of patients hospitalized with atrial fibrillation with thromboembolic risk factors (CHA_2DS_2VASc score >0 in men and >1 in women) stratified by sex and prescription of oral anticoagulation

	Overall	Men		Women	
		Prescribed anticoagulation	No anticoagulation	Prescribed anticoagulation	No anticoagulation
**Number of patients**	161240	45093	36868	39671	39608
**Events at 30 days**					
Myocardial infarction	6846 (4.2)	1519 (3.4)	2373 (6.4)	954 (2.4)	2000 (5.0)
Ischemic stroke	6107 (3.8)	1637 (3.6)	1136 (3.1)	1635 (4.1)	1699 (4.3)
Major bleeding	3329 (2.1)	858 (1.9)	974 (2.6)	587 (1.5)	910 (2.3)
Cardiac death	5328 (3.3)	655 (1.5)	1753 (4.8)	606 (1.5)	2314 (5.8)
All-cause death	12148 (7.5)	1321 (2.9)	4253 (11.5)	1139 (2.9)	5435 (13.7)
MACE	14354 (8.9)	1970 (4.4)	4801 (13.0)	1669 (4.2)	5914 (14.9)
**Events at 1 year**					
Myocardial infarction	10165 (6.3)	2444 (5.4)	3268 (8.9)	1612 (4.1)	2841 (7.2)
Ischemic stroke	9936 (6.2)	2323 (5.2)	2035 (5.5)	2470 (6.2)	3108 (7.8)
Major bleeding	7524 (4.7)	2116 (4.7)	1935 (5.2)	1732 (4.4)	1741 (4.4)
Cardiac death	16146 (10.0)	2979 (6.6)	4368 (11.8)	2916 (7.4)	5883 (14.9)
All-cause death	38213 (23.7)	6135 (13.6)	11950 (32.4)	5723 (14.4)	14405 (36.4)
MACE	42513 (26.4)	7395 (16.4)	12977 (35.2)	6761 (17.0)	15380 (38.8)

Figure S1. Trends in oral anticoagulation therapy for patients admitted to hospital with non-valvular atrial fibrillation with thromboembolic risk factors (CHA_2DS_2VASc score >0 in men and >1 in women)

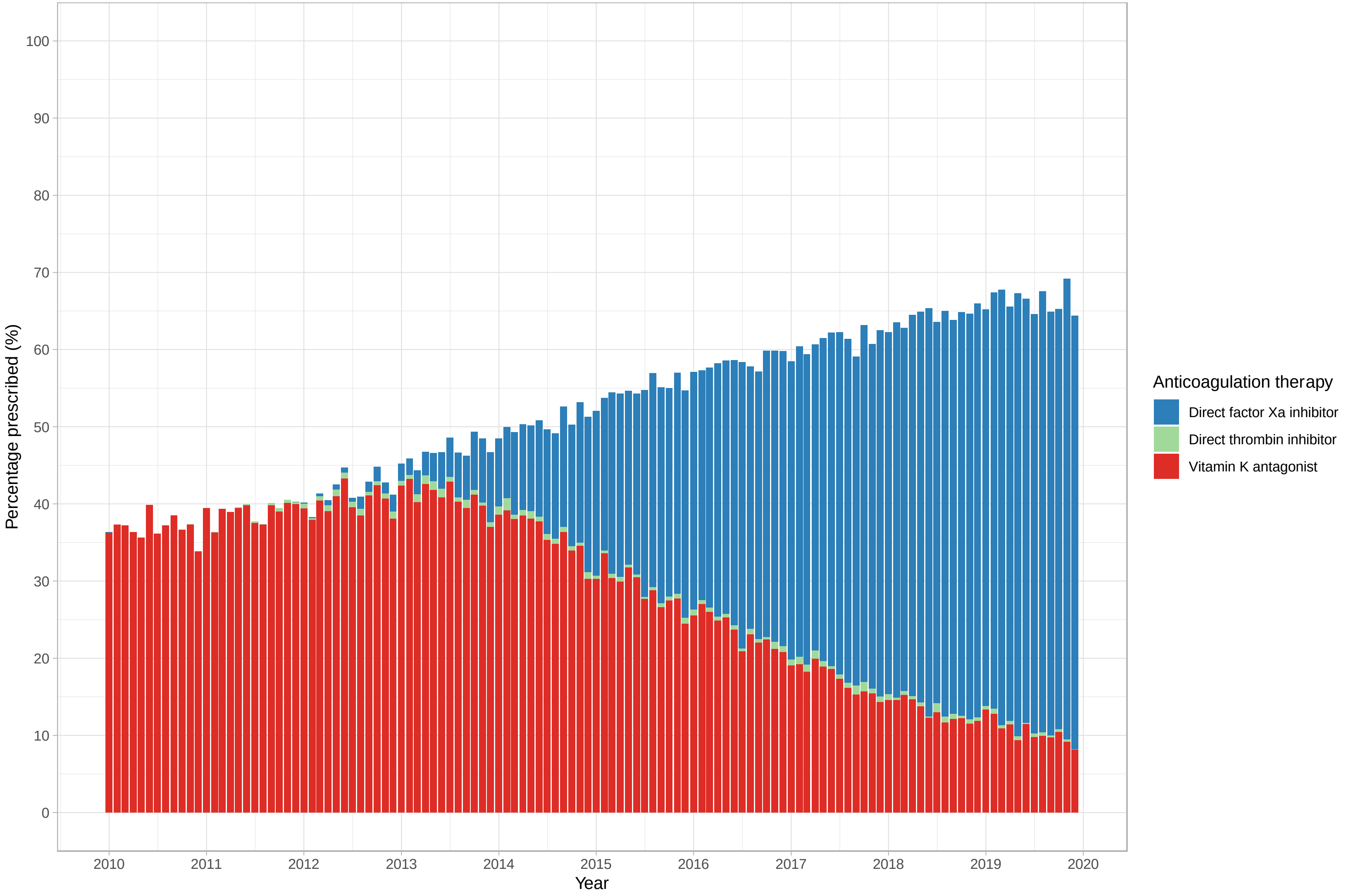

Figure S2. Trends in oral anticoagulation therapy for patients admitted to hospital with non-valvular atrial fibrillation with thromboembolic risk factors (CHA_2DS_2VASc score >0 in men and >1 in women) stratified by sex

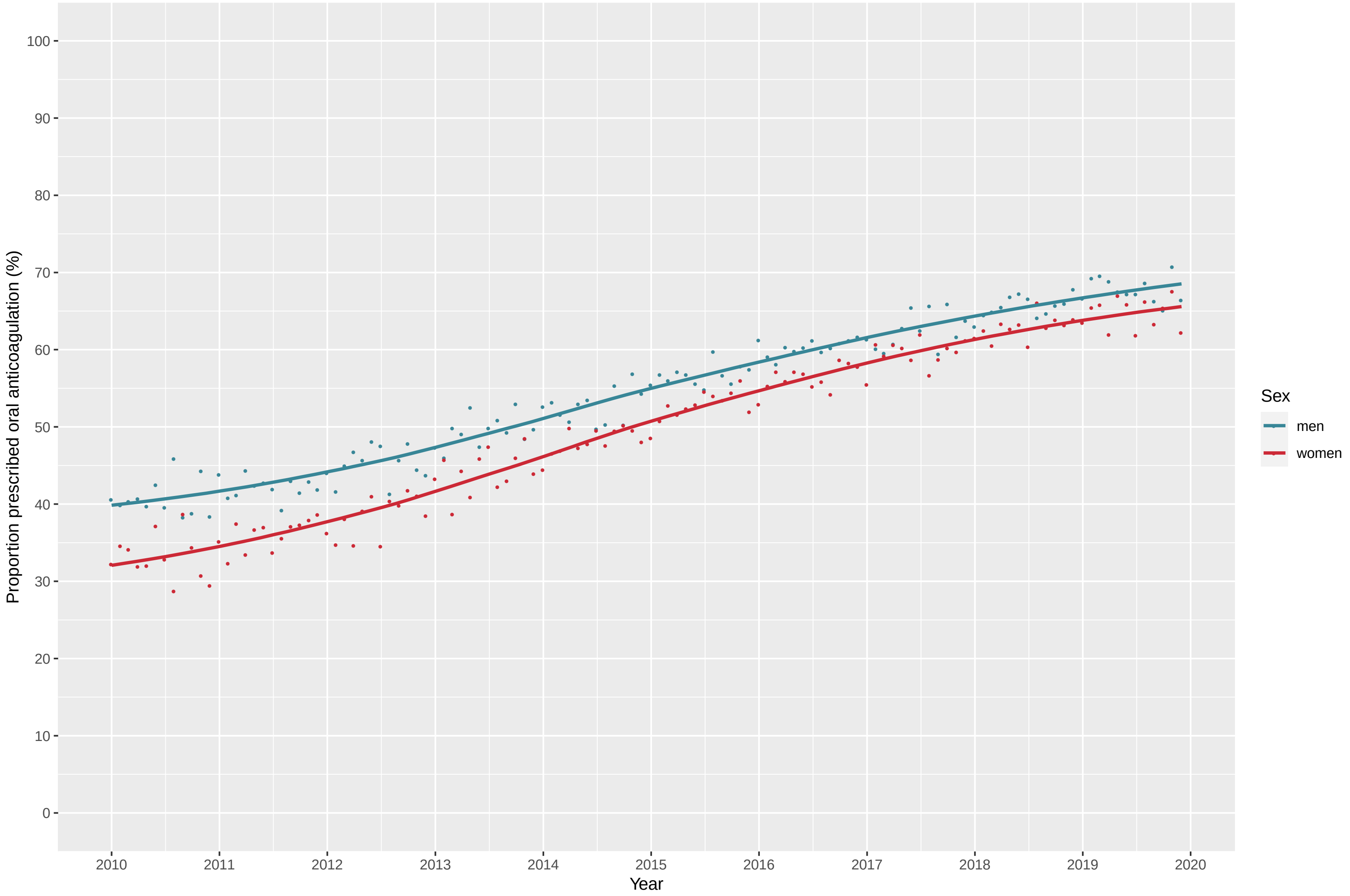

Figure S3. Factors associated with vitamin K antagonist and factor Xa inhibitor therapy in patients admitted to hospital with non-valvular atrial fibrillation with thromboembolic risk factors (CHA2DS2VASc score >0 in men and >1 in women).

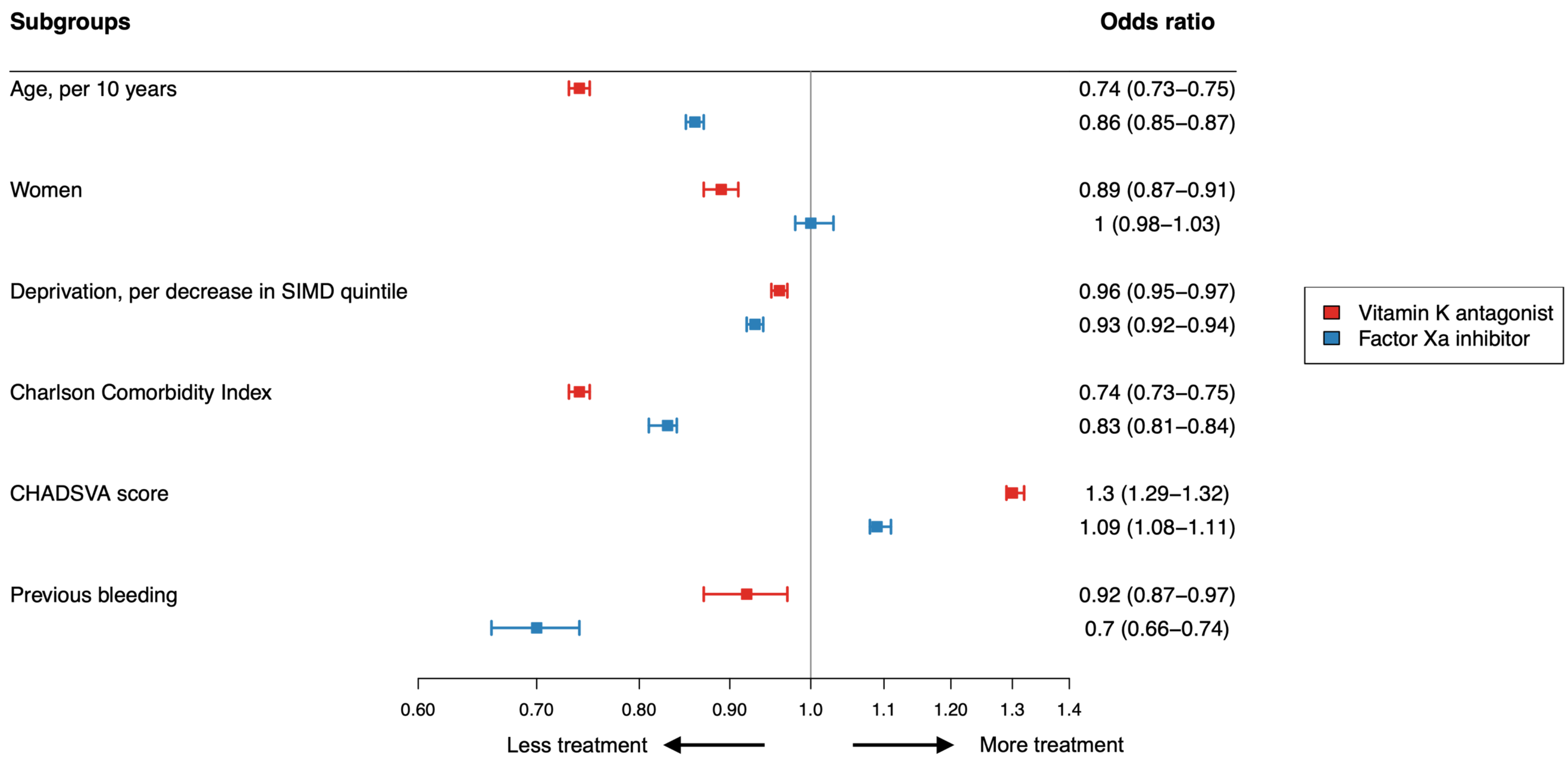

Figure S4. Cumulative incidence functions of outcomes of patients admitted to hospital with non-valvular atrial fibrillation with thromboembolic risk factors (CHA_2DS_2VASc score >0 in men and >1 in women) stratified by sex and prescription of oral anticoagulation therapy

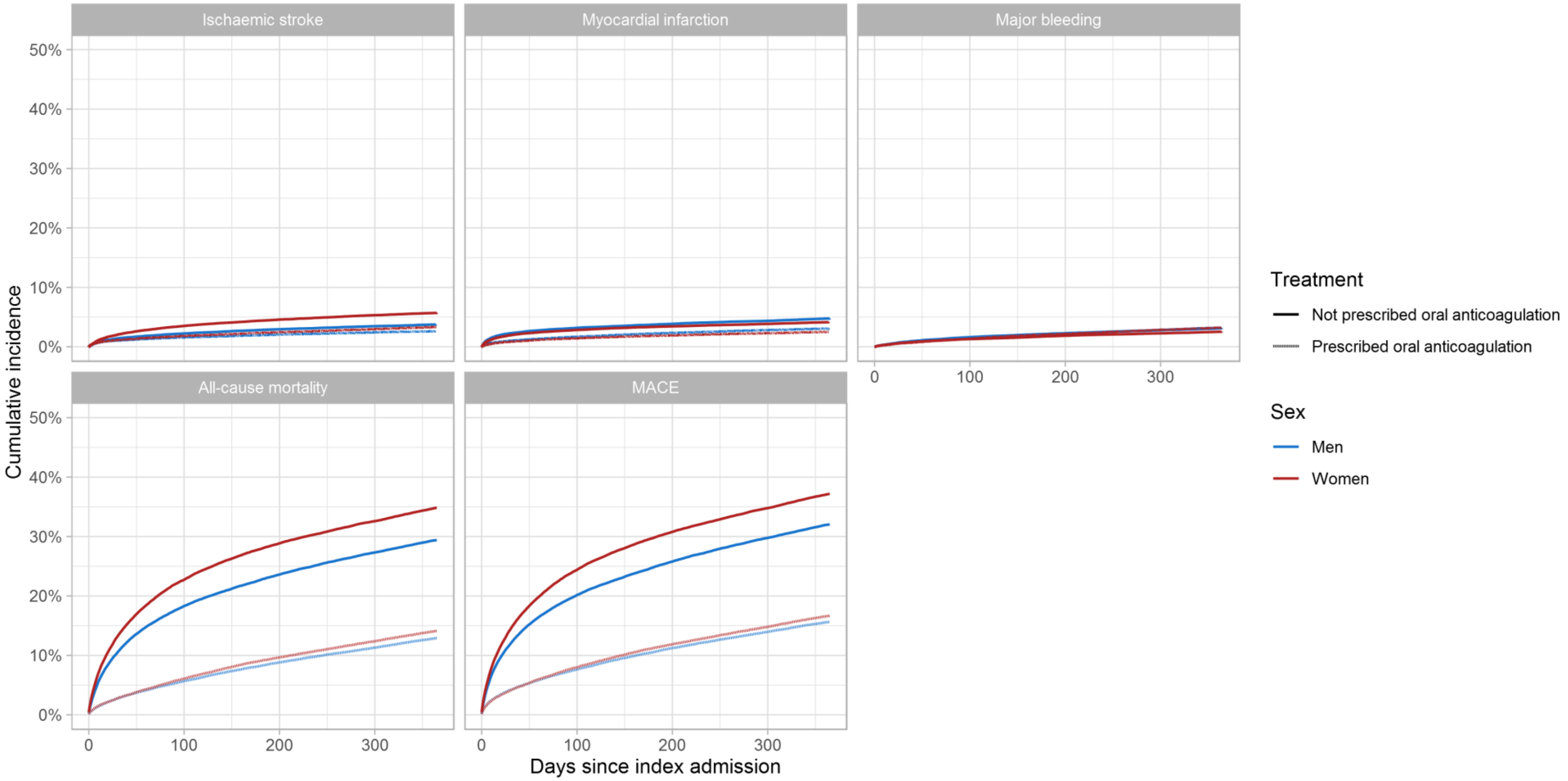

Supplement: Supplementary file 1 — Data S1–S3 Tables S1–S2 Figures S1–S4 [file JAH3-12-e027211-s001.pdf]
